# Simulations of Femtosecond-Laser Near-Field Ablation Using Nanosphere under Dynamic Excitation

**DOI:** 10.3390/ma17153626

**Published:** 2024-07-23

**Authors:** Jiaxin Sun, Lan Jiang, Mingle Guan, Jiangfeng Liu, Sumei Wang, Weihua Zhu

**Affiliations:** 1 Laser Micro/Nano Fabrication Laboratory, School of Mechanical Engineering, Beijing Institute of Technology, Beijing 100081, China; 3120170253@bit.edu.cn (J.S.); jianglan@bit.edu.cn (L.J.); 3220220246@bit.edu.cn (M.G.); 3120220365@bit.edu.cn (J.L.); 3120170251@bit.edu.cn (W.Z.); 2 Yangtze Delta Region Academy, Beijing Institute of Technology, Jiaxing 314019, China; 3 Beijing Institute of Technology Chongqing Innovation Center, Chongqing 401120, China

**Keywords:** femtosecond laser, simulation, intrapulse feedback, FDTD, plasma, TTM

## Abstract

Femtosecond lasers have garnered widespread attention owing to their subdiffraction processing capabilities. However, their intricate natures, involving intrapulse feedbacks between transient material excitation and laser propagation, often present significant challenges for near-field ablation predictions and simulations. To address these challenges, the current study introduces an improved finite-difference time-domain method (FDTD)–plasma model (plasma)–two-temperature model (TTM) framework for simulating the ablation processes of various nanospheres on diverse substrates, particularly in scenarios wherein dynamic and heterogeneous excitations significantly influence optical-field distributions. Initially, FDTD simulations of a single Au nanosphere on a Si substrate reveal that, with transitions in the excitation states of the substrate, the field-intensity distribution transforms from a profile with a single central peak to a bimodal structure, consistent with experimental reports. Subsequently, simulations of a polystyrene nanosphere array on a SiO_2_ substrate reveal that different excitation states of the nanospheres yield two distinct modes, namely near-field enhancement and masking. These modes cannot be adequately modeled in the FDTD simulations. Our combined model also considers the intrapulse feedback between the electromagnetic-field distribution resulting from near-field effects and material excitations. Furthermore, the model can quantitatively analyze subsequent electron–phonon coupling and material removal processes resulting from thermal-phase transitions. Consequently, our model facilitates predictions of the femtosecond-laser ablation of single nanospheres or nanosphere arrays with varying sizes and materials placed on substrates subjected to near-field effects.

## 1. Introduction

Femtosecond-laser processing boasts remarkable characteristics of high intensity and precision, facilitating the machining of diverse materials. However, optical diffraction imposes constraints on femtosecond lasers, hindering the direct processing of subwavelength structures. In this regard, surface nanospheres, which can circumvent optical diffraction limits by redistributing the optical fields generated by near-field effects, emerge as promising solutions, finding unique applications in femtosecond-laser processing [1,2,3]. In particular, large arrays of regularly patterned nanospheres facilitate the efficient and cost-effective fabrication of periodic structures [4,5].

However, the intricacies of light propagation at the micro and nano scales, as well as the transient changes observed in the optical properties of nonmetallic materials subjected to femtosecond-laser irradiation, pose challenges in simulating laser–material interactions driven by near-field effects [6,7,8,9]. Various simulation methods have been proposed to forecast the optical-field distributions resulting from near-field effects. In particular, the theory of Mie scattering has been demonstrated to effectively capture the light-scattering phenomena originating from individual nanoparticles [10]. Meanwhile, the FDTD has been reported to be suitable for modeling the interactions between nanospheres [11], thus predicting electric field-intensity distributions. However, these approaches assume ground-state optical properties for materials, thus yielding inaccurate predictions of distributions, particularly when dealing with nonmetallic materials. The properties of excited silicon are considered through the Sipe–Drude model [12] in predicting the generation of laser-induced periodic surface structures. Similarly, the field-intensity distribution caused by the dynamic excitation of microspheres is calculated using FDTD simulations with metallic hemispheres [13]. Different excitation states correspond to different field-intensity distributions and these varying field-intensity distributions, in turn, affect the material’s excitation rate. Therefore, in simulating the near-field ablation of nonmetallic materials, it is essential to consider the coupling between material excitation and field-intensity distribution, known as intrapulse feedback [14].

Furthermore, integrating the TTM in the simulation of laser–material interactions is essential for predicting the removal of metals and semiconductors. This is because the thermal phase transition resulting from electron–phonon coupling may dominate the subsequent material removal process [15]. Additionally, due to near-field effects, the source term in the TTM also depends on the previously mentioned field-intensity distributions. Some standard models based on plasma–TTM consider the transient optical changes in materials under laser irradiation. However, since the prediction of the laser distribution is simplified to surface reflection following Fresnel’s theorem and internal absorption following Beer’s law, they are not suitable for processes involving diffraction and scattering in the interaction between electromagnetic waves and micro/nano structures.

Therefore, in the simulation of femtosecond-laser near-field ablation using nanospheres, it is crucial to simultaneously consider the field-intensity distributions, electronic excitation, and electron–phonon coupling processes to accurately predict the resulting ablation morphology. To this end, the current study developed a model integrating the FDTD, plasma, and TTM. Establishing this model helps to explain the differences in the field intensity distribution under varying laser fluences and material excitation states in existing models, demonstrating the importance of considering transient excitation. This combined model was used to simulate dual-peak ablation induced by a single Au nanosphere on a Si surface. The obtained numerical results demonstrated that the transient responses of semiconductors under femtosecond-laser irradiation significantly alter optical-field distributions. Then, the model was subsequently employed to simulate femtosecond-laser ablation in the presence of polystyrene (PS) nanosphere arrays on SiO_2_ surfaces, demonstrating the complex behaviors of dielectric materials under femtosecond-laser near-field ablation. Variations in laser fluences yielded two distinct excitation and ablation modes, namely near-field enhancement and masking, offering possibilities for diverse ablation morphologies. The model takes into account the dynamic optical properties of nonmetals and the thermal phase transition processes of non-dielectric materials, thus providing flexible simulation capabilities applicable to the near-field ablation of various materials.

## 2. Simulation Framework

**Figure 1:**
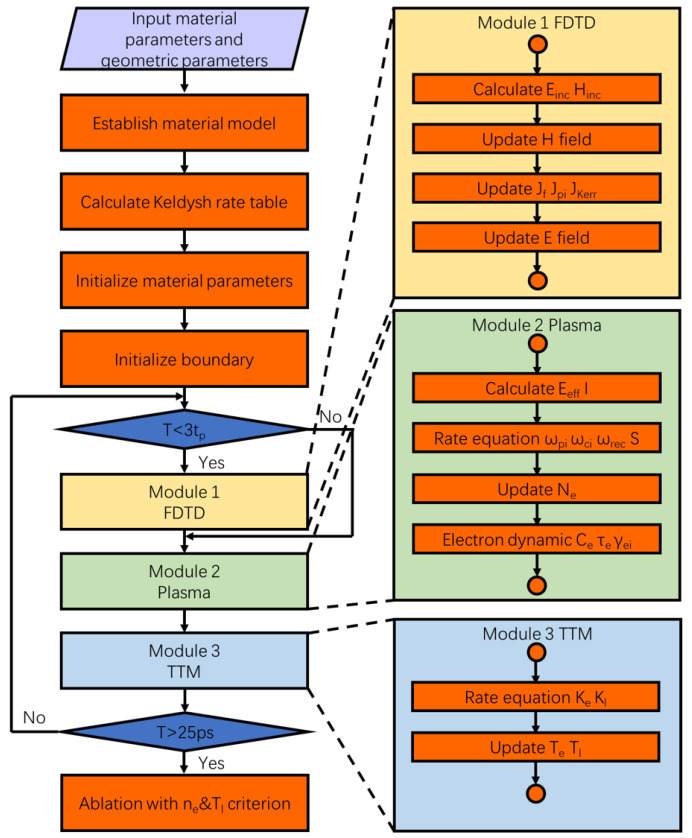
Flowchart illustrating the FDTD–plasma–TTM model. The three modules interact by exchanging parameters, and detailed definitions and parameter calculations are summarized in the next three subsections.

Although the near-field ablation process of femtosecond lasers generally involves numerous temporal and spatial scales, the underlying physical processes can typically be categorized into three components: (a) the propagation of the femtosecond laser, modeled using the FDTD; (b) transient excitation of the electronic system of the material, computed using the plasma; and (c) subsequent electron–phonon coupling and thermal/nonthermal ablation of the material, assessed using the TTM. Within the simulation framework, these components correspond to three modules, with computational processes as depicted in Figure 1. The simulation program was written using MATLAB 2020b and was run on a single NVIDIA Quadro RTX-5000 GPU for higher computational efficiency (NVIDIA Corporation, Santa Clara, CA, USA).

Figure 1 depicts two crucial time nodes: three times the pulse width (t_*p*_) and 25 ps. To accurately capture the complete temporal distribution of Gaussian-type femtosecond-laser incidence, the pulse was timed to reach its peak at 1.5 times t_*p*_, ensuring that the majority of the laser energy was concentrated within three times t_*p*_. Furthermore, the maximum simulation duration was set to 25 ps, which surpasses the typical material electron–phonon coupling time constant (10 ps). Notably, once the electron–phonon temperature approaches equilibrium, the temperature will continue to decrease owing to thermal diffusion. To manage the substantial computational workload of the 3D model, the Keldysh rate of the involved materials was precomputed, and $w_{\mathrm{pi}}$ was determined through interpolation. The above flowchart illustrates that the propagation of the laser is influenced by the excitation of the material, and that the resulting electric-intensity further excites the material. Notably, when nonmetallic materials experience significant changes in their optical properties under femtosecond-laser irradiation, the electric-intensity distributions in the materials undergo dynamic changes under intrapulse feedback. Relevant detailed explanations will be provided in the subsequent subsections.

### 2.1. Finite-Difference Time-Domain Method

Notably, the model describing the propagation of femtosecond lasers and the optical response of materials is grounded within 3D nonlinear Maxwell’s equations [16]. These can be solved using the auxiliary differential equation method [17], as presented below:

(1) $$\frac{\partial \vec{E}}{\partial t}=\frac{\nabla \times \vec{H}}{\varepsilon_{0}\varepsilon_{\infty }}-\frac{1}{\varepsilon_{0}\varepsilon_{\infty }}\left({\vec{J}}_{f}+{\vec{J}}_{pi}+{\vec{J}}_{Kerr}\right),$$

(2) $$\frac{\partial \vec{H}}{\partial t}=-\frac{\nabla \times \vec{E}}{\mu_{0}},$$

(3) $$\frac{\partial {\vec{J}}_{f}}{\partial t}=-\frac{{\vec{J}}_{f}}{\tau_{e}}+\frac{e^{2}n_{e}}{m_{e}}\vec{E},$$

(4) $${\vec{J}}_{pi}=E_{g}\frac{w_{\mathrm{pi}}\vec{E}}{{\left|\vec{E}\right|}^{2}}\frac{n_{v}-n_{e}}{n_{v}},$$

(5) $${\vec{J}}_{Kerr}=\epsilon_{0}\chi_{3}\frac{\partial \left({\left|\vec{E}\right|}^{2}\vec{E}\right)}{\partial t},$$

where $\vec{E}$, $\vec{H}$, and $\vec{J}$ represent the electric field, magnetic field, and equivalent current induced by the material’s response, respectively; $\varepsilon_{0}$ and $\mu_{0}$ denote the permittivity and permeability of free space, respectively; and $\varepsilon_{\infty }$ indicates the permittivity of the material, which has a value of 1 for metals and can be computed using the ground-state dielectric function for other materials. Furthermore, the term ${\vec{J}}_{f}$ represents the acceleration and scattering processes of free electrons in materials subjected to an electric field, as derived from the Drude model, $m_{e}$ is the mass of the electron, and $\tau_{e}$ is the electron relaxation time. Moreover, the term ${\vec{J}}_{pi}$ describes the electric-field loss incurred when overcoming bandgap $E_{g}$ with a rate $w_{\mathrm{pi}}$, assuming the absence of ionization processes in metals.The introduction of the initial electron density in the valence band $n_{v}$ is intended to correct the ionization rate of the material near saturation, and the corresponding value for the material is provided in the plasma. Meanwhile, the term ${\vec{J}}_{Kerr}$ describes the Kerr polarization effect, computed using the third-order susceptibility $\chi_{3}$, which is crucial for delineating self-focusing effects in SiO_2_ but can be disregarded for other materials considered herein. The material parameters used in the FDTD are provided in Table 1. However, for a comprehensive description of laser–material interactions, further characterizations of material excitation in plasma are essential.

### 2.2. Plasma Model

The plasma frequencies of materials are crucial for material excitation computations based on the Drude model. Furthermore, the processes leading to variations in the free-electron density include photoionization ($w_{\mathrm{pi}}$), collisional ionization ($w_{\mathrm{ci}}$), carrier recombination ($w_{\mathrm{rec}}$), and carrier migration (which is disregarded here). Therefore, for nonmetallic materials, the fluctuation in electron density can be modeled as follows:

(6) $$\frac{\partial n_{e}}{\partial t}=\left(w_{\mathrm{pi}}+w_{\mathrm{ci}}\right)\left(1-n_{e}/n_{v}\right)-w_{rec}.$$

Equations and parameters governing ionization processes in SiO_2_ [21,22], PS [20,23], and Si [24,25] can be referenced from the relevant literature. Meanwhile, for circular polarization, the rate of photoionization is set to half of that for linear polarization. Notably, in the FDTD method, the transient electric intensity cannot be directly employed to compute ionization rates. Instead, the envelope of the electric field, E_*eff*_, must be utilized to derive the optical intensity, *I*, and compute photoionization and collision ionization rates. In addition to the electron density of the material, the energy deposited in the free electron system should be taken into consideration and further be employed in the TTM. The process of varying the energy can be modeled as follows:

(7) $$S={\vec{J}}_{f}\cdot \vec{E}-E_{g}w_{\mathrm{ci}},$$

where $-E_{g}w_{\mathrm{ci}}$ signifies the decrease in free electron energy caused by collisional ionization, which is irrelevant for metals. For Si, computations of the increase in the internal energy of the electron system must consider processes such as single-photon absorption, two-photon absorption, and free-electron absorption, as described by Equation (12) of another study [25]. The plasma model, combined with the aforementioned FDTD method, can elucidate laser–material interaction processes. The material parameters used in the plasma model are provided in Table 2.

### 2.3. Two-Temperature Model

Despite the occurrence of the intrapulse feedback phenomenon during femtosecond-laser irradiation, the process of electron–phonon coupling can be neglected. Notably, in metal and semiconductor ablation, thermal ablation predominates as the primary mechanism of material removal. Hence, determining lattice temperatures based on the TTM can aid in deriving the final ablation morphology. The TTM, derived from the plasma model to determine the electron relaxation duration, electron–phonon coupling coefficient, and source, can be expressed [28] as follows:

(8) $$C_{e}\frac{\partial T_{e}}{\partial t}=\nabla \left(k_{e}\nabla T_{e}\right)-G\left(T_{e}-T_{l}\right)+S,$$

(9) $$C_{l}\frac{\partial T_{l}}{\partial t}=\nabla \left(k_{l}\nabla T_{l}\right)+G\left(T_{e}-T_{l}\right),$$

where $k_{e}$, $C_{e}$, $k_{l}$, and $C_{l}$ denote the thermal diffusion and specific heats of electrons and phonons, respectively, while *G* represents the electron–phonon coupling coefficient of the material. For femtosecond lasers, the source term S depends on the optical distribution of the femtosecond laser, while calculations of the electron thermal diffusion, ke, and heat capacity, $C_{e}$, rely on the electron density and temperature. Consequently, after termination of laser action, electron-density reduction resulting from electron recombination and temperature reductions caused by thermal diffusion and electron–phonon coupling must also be computed based on the plasma model. The material parameters used in the TTM are provided in Table 3. For dielectric materials, the criterion for material removal in the model depends on the free electron density. Therefore, the TTM can be disregarded in calculations for SiO_2_ and PS.

## 3. Result and Discussion

### 3.1. Single Au Nanosphere on a Si Substrate Subjected to Linearly Polarized Laser Irradiation

**Figure 2:**
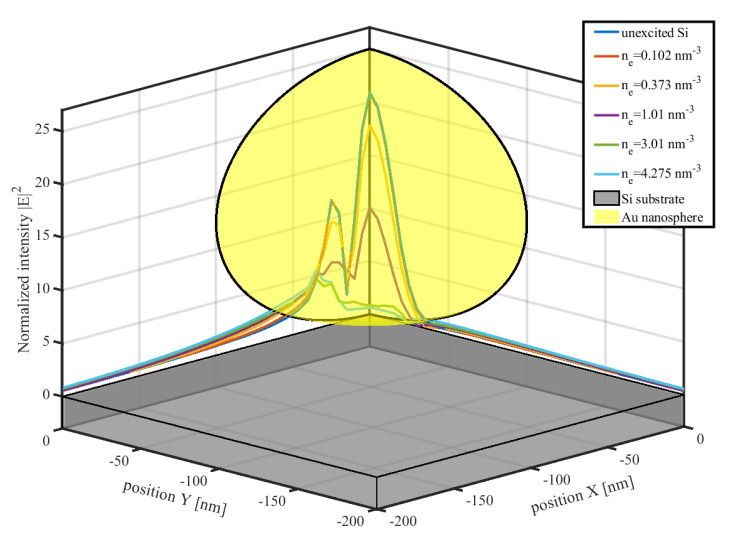
Illustrations of optical-field distributions within the Si substrate under varying Si excitation levels. The normalized intensity variation along the X and Y axes at Z = −5 nm is shown. The electron relaxation time considered by the Drude model is fixed at 1.1 fs for Si [30]; hence, the excitation and corresponding optical property are only dependent on electron density $n_{e}$.

Here, we present the femtosecond-laser ablation simulation of a 200 nm Au nanosphere on a Si substrate as an illustrative example. Interestingly, the FDTD method offers versatility as it is applicable across diverse particle shapes and arrangements, and considers interactions between particles and substrates. However, when materials are subjected to femtosecond-laser irradiation, their optical properties vary. Considerations of the electromagnetic-field distributions of the materials under varying excitation states represent possible solutions to this concern. According to the Drude model, varying electron densities yield different dielectric functions for Si substrates, thus altering the electric-intensity distribution. Figure 2 presents the optical-field distributions under different excitation levels simulated using commercial FDTD software (FDTD Solutions 2020 R2, Lumerical Inc., Vancouver, BC, Canada). Notably, in these FDTD simulations, plane waves linearly polarized along the X-direction are employed.

The electric-field distributions depicted in Figure 2 exhibit significant variations with changing electron densities of the Si substrate. Along the Y-direction, increasing electron densities lead to decreasing electric-field intensities. Conversely, the intensity distribution along the X-direction displays a more complex variation trend. At low electron densities, a central peak surrounded by other peaks is apparent. In contrast, at higher electron densities, the center peak disappears.

**Figure 3:**
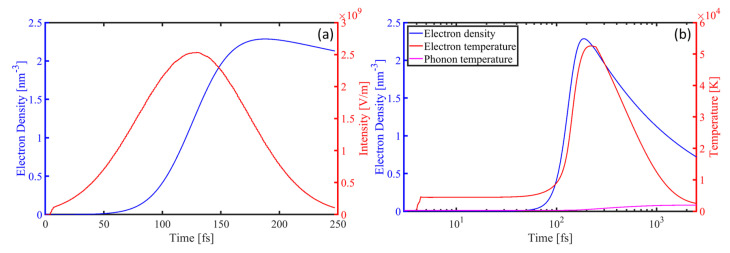
(**a**) Temporal evolutions of electric intensity E_*x*_ (normalized to the maximum) and electron density $n_{e}$ at z = −5 nm in center. (**b**) Temporal evolutions of the electron density, electron temperature, and phonon temperature. The time axis adopts a logarithmic scale to enhance visualizations of the electron–phonon coupling process.

While similar methods are often employed to approximate the electric-intensity distributions of excitable materials, material excitation processes are inherently dynamic and heterogeneous. Thus, we employed the FDTD–plasma–TTM model, which considers intrapulse feedback, to simulate the excitation and ablation processes. Specifically, a single Au nanosphere on the surface of a Si substrate was irradiated by a linearly polarized femtosecond laser (800 nm, 80 fs, and 0.4 J/cm²), and the beam waist diameter was 2 μm. Figure 3 illustrates the temporal evolution of the excitation states of Si at z = −5 nm in the center, including curves depicting the external electric field, electron density, electron temperature, and phonon temperature. Notably, the simulation duration of the FDTD ranges from −1.5 t_*p*_ to 1.5 t_*p*_, totaling 240 fs, while that of the TTM extends to 25 ps. A grid size of 10 nm × 10 nm × 10 nm was employed.

**Figure 4:**
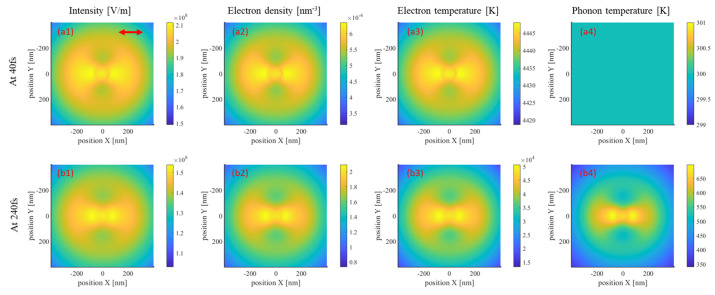
Effective electric field, transient electron density, temperature, and phonon temperature maps at (**a1**–**a4**) 40 fs and (**b1**–**b4**) 240 fs in depth of z = −5 nm. The electric intensity corresponds to its effective value over one cycle rather than its instantaneous value.

As depicted in Figure 3a, the electron density within the plasma model exhibits significant variations. For the pulse peaking at 120 fs, no noticeable variations can be observed in the electron density before 50 fs. This is because the initial increase in electron density primarily originates from photoionization, while the rate of collisional ionization depends on the density of free electrons, which is initially inadequate. However, during the subsequent 100 fs, the electron density significantly increases under laser irradiation, surpassing the critical free electron density and resulting in the metallization of the Si material. Throughout this period, changes in the excitation state alter the optical-field distribution, inducing the intrapulse feedback phenomenon. In the following period, Auger recombination in Si predominantly contributes to the reduction in electron density, which is crucial for TTM computations, particularly those concerning the electron specific heat and electron–phonon coupling coefficients. Figure 3b illustrates the changes in electron density, electron temperature, and phonon temperature during the electron–phonon coupling process, lasting approximately 10 ps. After 180 fs, the rate of recombination overpowers the rate of free-electron generation, reducing the electron density. By 240 fs, which is three times t_*p*_, assuming the termination of laser action, only recombination processes persist. Meanwhile, the electron temperature rapidly increases to 4300 K and remains constant over a period, consistent with reports in the literature [25]. At this stage, the material primarily absorbs laser light through single-photon absorption. Photon energies exceeding the bandgap contribute to an increase in the electron density, and the temperature is maintained below 4380 K. With further increments in the electric intensity and electron density, two-photon absorption and free-electron absorption both lead to a rapid increase in the electron temperature. Subsequently, Auger recombination and electron–phonon coupling lead to a decrease in the electron density and temperature, as well as an increase in the phonon temperature. These variations occur relatively gradually compared to the transient changes observed in the plasma model during laser irradiation. Thus, Figure 3, depicting the temporal evolution of material excitation characteristics, underscores the importance of employing the FDTD–plasma–TTM model. However, in addition to these factors, spatial variations in electric-intensity distributions induced by near-field effects also need consideration. Figure 4 presents maps of the electron density, electron temperature, and phonon temperature within the Si substrate at 40 fs and 240 fs. As the wavefront is distorted by the nanosphere, the transient electric intensity cannot directly reflect the intensity distribution. Instead, the effective field strength, representing the average value over one optical cycle, is presented here.

**Figure 5:**
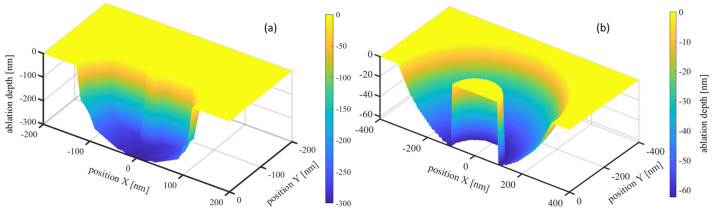
(**a**) Ablation morphology of the Si substrate derived from FDTD–plasma–TTM model. (**b**) Ablation morphology of the Si substrate derived from standard plasma–TTM model. Notably, the simulation records the maximum phonon temperature, and regions exceeding 1689 K [31] are removed.

In Figure 4, the snapshots at (a) 40 fs and (b) 240 fs, respectively, correspond to the ground and excited states of the material. At 40 fs, the spatial-intensity distribution depicted in (a1) resembles that corresponding to the unexcited Si substrate computed using the FDTD method, as displayed in Figure 2. Meanwhile, in the spatial-intensity distribution depicted in (b1), the central peak is comparatively less prominent than the peaks on either side. Additionally, the electron-density distribution mirrors the intensity pattern. In the electron temperature distribution profile depicted in (a3), most regions are concentrated around 4300 K owing to previously stated reasons. Nevertheless, following excitation, the electron temperature of the material reaches a relatively high level, as depicted in (b3). Because the electron–phonon coupling process is significantly slower than electron excitation, the phonon temperature depicted in (a4) remains at 300 K, still significantly lower than the electron temperature displayed in (b4). However, to ascertain the final ablation morphology, the plasma model and the TTM must be employed to analyze the subsequent electro-thermal coupling processes. Figure 5a presents the ablation morphology predicted based on temperature criteria. Remarkably, the simulation yielded ablation results resembling experimental outcomes [30], displaying an elliptical shape with a major axis of approximately 160 nm.

For comparison, a standard plasma–TTM model was used to simulate the process of laser irradiation on Au nanospheres with a Si substrate. In the standard model, the intensity of the laser depends on the transient incident laser intensity, the reflectivity, and the absorptivity of the material along the propagation path. When considering the additional gold–air interface, the laser intensity undergoes significant attenuation due to the high reflectivity of gold. At the same time, based on the thickness of the gold nanosphere at different radial distances, the laser intensity reaching the Si material after absorption can sharply decrease. Using the standard model, the ablation result is shown in Figure 5b.

In Figure 5b, the ablation area is ring-shaped. Due to the assumption of vertical downward laser propagation, the Au in the central region strongly absorbs the laser, resulting in no ablation in the area beneath the nanosphere. Meanwhile, outside the central region, the ring-shaped area undergoes ablation due to reaching the melting temperature, influenced by the Gaussian intensity distribution of the beam. Since the standard model does not include the polarization characteristics of the laser and only considers the particle nature of the femtosecond laser, the ablation morphology lacks the polarization dependence observed in Figure 5a. Clearly, the standard model is inadequate for explaining the near-field enhancement effects of the nanosphere and predicting the ablation for different polarization laser incidences.

### 3.2. PS Nanosphere Array on a SiO_2_ Substrate Subjected to Circularly Polarized Laser Irradiation

**Figure 6:**
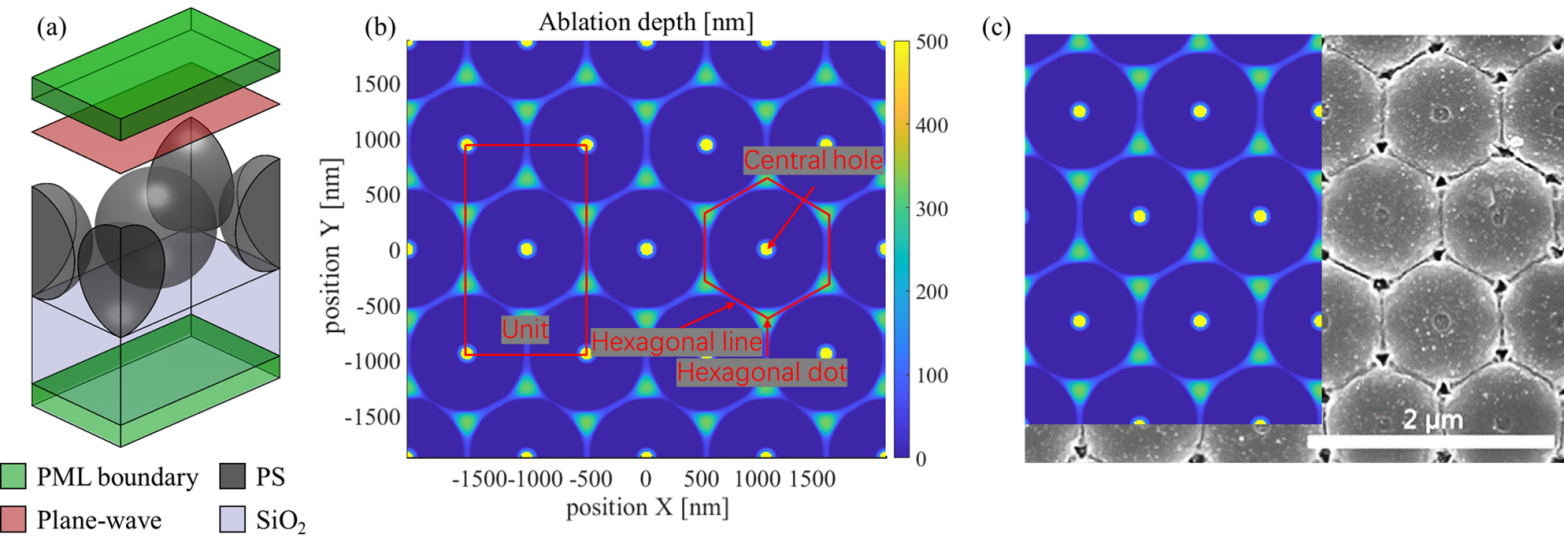
(**a**) Schematic of the simulation unit. The four PBCs are not depicted to better illustrate the internal structure. (**b**) Ablation result for an area obtained by repeating the unit in (**a**) under a fluence of 3.02 J/cm². (**c**) Comparison of simulation result with experiment result from previous work [32], with laser parameters of fluence of 2.69 J/cm², pulse duration of 35 fs, and center wavelength of 800 nm.

Through the previous simulation involving a single Au nanosphere on a Si substrate, we demonstrated the dynamic distribution of the electromagnetic field resulting from intrapulse feedback. In comparison, a simulation involving the femtosecond-laser irradiation of a PS nanosphere array on a SiO_2_ substrate is anticipated to be more complex. Typically, the optical responses of dielectric materials exhibit a broad range of variations, from transparency in the ground state to high reflectivity and absorption in the excited state. Concurrently, interactions between nanospheres in the array can be expected to result in an optical-field distribution that is different from that for a single nanosphere. Hence, to accurately model this ablation, the FDTD for electromagnetic field propagation must be coupled with the plasma model for material excitation. Previous studies [13] have reported variations in ablation modes depending on laser fluence. For instance, a PS array on a soda lime glass substrate has been reported to demonstrate central hole ablation at low fluences and hexagonal dot and line ablations at high fluences. Conversely, the ablation results [32] for a PS array on a SiO_2_ substrate demonstrate the potential coexistence of central ablation and hexagonal spot and line ablation. Notably, intensity predictions based on the FDTD are unable to account for the above two ablation modes. Conversely, the FDTD–plasma model, which integrates both laser propagation and material excitation, can adequately simulate different ablation modes within a unified framework. Figure 6 presents (a) the individual unit employed in the simulation, as well as (b) the simulation results for an ablation area subjected to a fluence of 3.02 J/cm². Notably, for the light source, the simulation employed a circularly polarized planewave with t_*p*_ of 35 fs, incident from the source plane located 400 nm away from the top of the nanospheres. The diameter of the PS nanospheres (gray) was set to 1 µm, while the separation distance between nanospheres was set to 40 nm to properly simulate the size errors and arrangement defects of the nanospheres. Below the nanospheres, a 1 µm thick layer of SiO_2_ was created (blue). Moreover, periodic boundary conditions (PBCs) were applied along the XY direction, while perfectly matched layers (PMLs) were applied along the Z direction.

**Figure 7:**
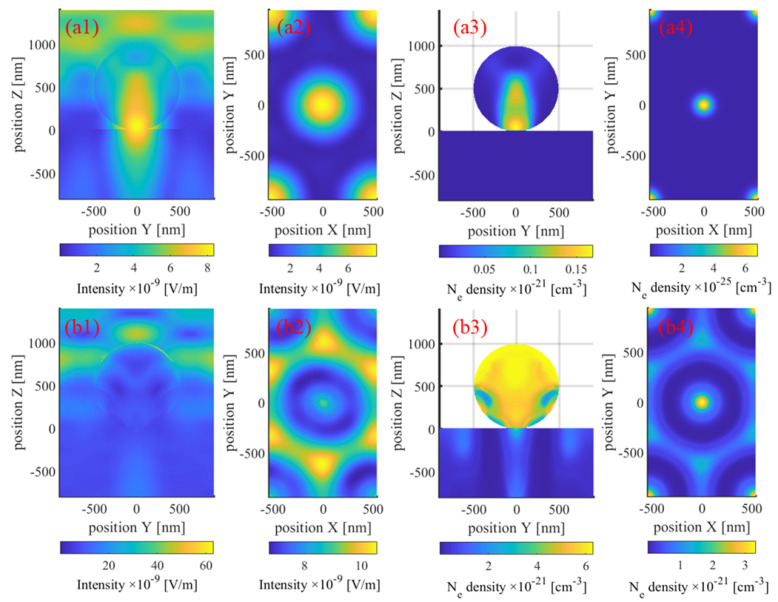
Effective electric-intensity distributions within the (**a1**) x = 0 nm and (**a2**) z = −20 nm planes and transient electron-density distributions within the (**a3**) x = 0 nm and (**a4**) z = −20 nm planes at 20 fs. The corresponding plots at 50 fs are depicted in (**b1**–**b4**). The laser fluence was 2.67 J/cm².

In Figure 6b, two distinct ablation modes are apparent. In particular, an ablation area appears directly below each nanosphere. Meanwhile, ablations at the gaps between nanospheres form hexagonal dot and line ablation areas. Notably, the ablation structures are generally consistent with those reported in the literature [32], which is shown in (c); however, in experiments, ablations of central holes tend to be weaker. This disparity can be explained based on material-removal differences between simulations and experiments. In simulations, regions, wherein the electron density exceeds the critical free electron density, are excluded, whereas, in experiments, material removal from central holes is obstructed by the PS nanospheres. To confirm that these central and hexagonal ablations indeed originate from different mechanisms, Figure 7 presents the electric-intensity and electron-density distributions at 20 fs and 50 fs.

**Figure 8:**
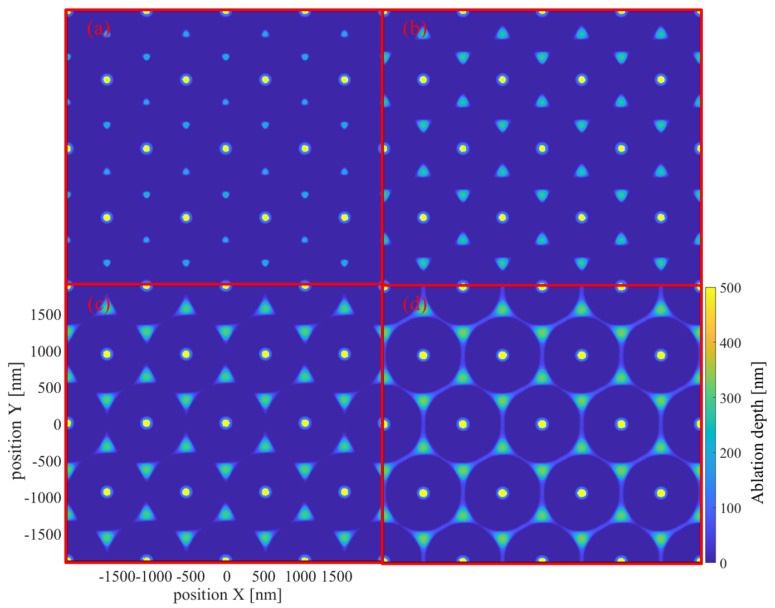
Ablation results at fluences of (**a**) 1.97, (**b**) 2.32, (**c**) 2.67, and (**d**) 3.02 J/cm², respectively.

At 20 fs, the optical properties of the material do not significantly differ from those in the ground state. Consequently, the field-intensity distributions depicted in (a1) and (a2) are consistent with those corresponding to near-field enhancement. The optical-field intensities at the bottom of the nanospheres and the top of the substrate are much higher than those in the hexagonal regions. In (a3), the electron density surrounding the nanospheres is significantly higher than that within the substrate, attributed to the considerably narrower bandgap of PS compared to that of SiO_2_. Owing to the superposition of near-field effects and nonlinear multiphoton ionization, the electron-density distribution at the center in (a4) is sharper compared to that in (a2). At 50 fs, the electric-intensity distribution of the excited material significantly changes. The electron densities within the PS nanospheres largely reach saturation; hence, the PS nanospheres serve as a mask. Metallization prevents the entry and convergence of the laser beam from the top, leading to the disappearance of near-field enhancement in (b1). However, owing to the presence of gaps in the nanosphere arrangements, a hexagonal region with high electric-intensity appears in (b2). Notably, the central hole with high electron density acts as a hot spot, as depicted in (b3), yielding the additional ring apparent at the center. Moreover, the electron-density distribution in (b4) closely agrees with that in the final ablation morphology. Evidently, the dynamic process of PS nanosphere excitation results in the dominance of two distinct effects, namely near-field enhancement and masking, which correspond to two optical field distributions and ablation modes. Although the central hole ablation may not be as prominent as the hexagonal ablation, the modification range of the material at the central holes may even exceed that at the hexagonal dots, as evidenced by the etching results displayed in Figure 3 of another study [32]. These distinct ablation mechanisms result in varying evolution patterns of the central hole and hexagonal ablation morphologies with increasing laser fluence, as depicted in Figure 8.

**Figure 9:**
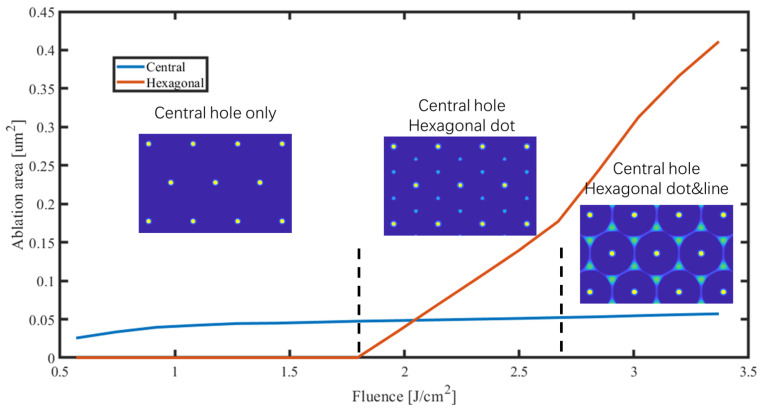
Variations in central and hexagonal ablation areas under different laser fluences. The laser fluence ranges from 0.57 J/cm² to 3.37 J/cm². Dashed lines are used to divide the laser fluence and the corresponding ablation results into three distinct regions. The colorbar in the ablation results of the illustrations is consistent with that in Figure 8.

For the central hole, the center region of the SiO_2_ substrate undergoes near-field enhancement during the initial stages under varying laser fluences. However, increased fluences accelerate the transition of PS nanospheres into masking states. Consequently, under varying laser fluences, the central area presents similar ablation sizes. Conversely, for hexagonal dots and lines, the PS nanospheres must be highly excited to produce the corresponding intensity distribution. Hence, increasing fluences enhance the ablation outcomes. Figure 9 illustrates the variations in the central and hexagonal ablation areas at a depth of 20 nm under a wider range of laser fluences. Based on differences in ablation locations, the profile can be divided into three stages, as depicted in Figure 9.

In Figure 9, a wider range of laser fluence is simulated, and the ablated area within the unit cell caused by two different mechanisms is quantified. In the first stage within the fluence range of 0.57 J/cm² to 1.62 J/cm², ablation is entirely dependent on near-field enhancement at the center. Due to the intense central enhancement, ablation occurs even lower than 0.57 J/cm². However, as the fluence increases, the ablation of central holes hardly expands. This may be due to two reasons: on the one hand, the dynamic excitation of PS nanospheres forms a “clamping” effect on the downward propagating laser, similar to the filamentation phenomenon [33,34]; on the other hand, the near-field enhancement is highly localized, and the ionization of SiO_2_ is also highly nonlinear; the increase in fluence has little effect on the area reaching the critical free-electron density. In the second stage, within the fluence range of 1.62 J/cm² to 2.67 J/cm², the laser intensity at the hexagonal dots is not subject to clamping due to the voids created by the PS nanosphere arrangement and, thus, it increases steadily with rising laser fluence. Additionally, the electric-intensity distribution does not exhibit extreme localization, as shown in Figure 7(a1,b1). In the third stage, within the fluence range of 2.67 J/cm² to 3.37 J/cm², additional ablation occurs along the hexagonal lines. In simulations involving metallic hemispheres [13], the electric-intensity distributions along the hexagonal lines are stronger than at the hexagonal points. However, in our simulation, under the mask formed at the highly excited tips of the microspheres, the electromagnetic field still needs to propagate a certain distance, which may lead to differences in the field strength distribution.

## 4. Conclusions

In this paper, we have developed a model that combines FDTD, plasma, and TTM to simulate the near-field ablation processes of various materials. Inclusion of intrapulse feedback, i.e., the coupling between light field propagation and transient excitation of the material, enables adaptation to the optical property variations over a wide range in nonmetallic materials, thereby yielding the distribution of energy deposition. Additionally, the dual-temperature model contributes to addressing material removal induced by thermal phase transitions. Subsequently, the model was applied to simulate a PS nanosphere array on a SiO_2_ substrate. Simulation of the ablation process at different fluences revealed that the same material and structure could exhibit two distinct modes of near-field enhancement or masking, depending on the excitation of the nanospheres. The improved model, tailored for near-field ablation processes influenced by intrapulse feedback, provides a novel perspective for mechanism exploration and result prediction. Our future work will aim to broaden the application scope of the model to simulate complex experimental conditions. This includes applying the model to scenarios involving oblique incident lasers on a nanosphere–substrate system and providing a theoretical explanation for the interference patterns caused by pairs of nanospheres at specific distances.

## Figures and Tables

**Table 1:** Material parameters for FDTD. For Au, the electron density and relaxation time are calculated using the plasma frequency and electron collision frequency from the literature [18]. The third-order nonlinearity [18] is not considered. For nonmetallic materials, the electron density calculation relies on the plasma.

Symbol	Unit	Au [18]	Si [19]	SiO_2_ [14]	PS [20]
$\varepsilon_{\infty }$	-	7.6	13.54	1	2
$\tau_{e}$	fs	0.0207	1	1	0.1
$n_{e}$	m^−3^	54.6499	dynamic	dynamic	dynamic
$\chi_{3}$	m^2^/V^2^	-	4 × 10^−20^	2 × 10^−22^	1 × 10^−22^

**Table 2:** Material parameters for plasma model. The collision ionization cross-section of PS is estimated to be 10 cm²/J. Since only processes within ultrashort pulses are considered, the recombination processes in SiO_2_ and PS are neglected. The maximum electron density of Si is assumed to be equal to the atomic density, and the maximum electron density of PS is assumed to be 6.31 × 10^27^ m^−3^, corresponding to the total density of styrene particles [26].

Symbol	Unit	Au	Si [25]	SiO_2_ [14]	PS [27]
$E_{g}$	eV	-	$E_{g}$($T_{l}$,$n_{e})$	9.0	4.05
$w_{\mathrm{pi}}$	m^−3^s^−1^	-	$w_{\mathrm{SPA}}+w_{\mathrm{TPA}}$	Keldysh	Keldysh
$w_{\mathrm{ci}}$	m^−3^s^−1^	-	$\sigma n_{e}$	$a_{c}In_{e}$	$a_{c}In_{e}$
$w_{\mathrm{rec}}$	m^−3^s^−1^	-	$\gamma n_{e}^{3}$	-	-
$n_{v}$	m^−3^	-	50 × 10^27^	22 × 10^27^	6.31 × 10^27^

**Table 3:** Material parameters for TTM. The carrier system of Si is assumed to be a non-degenerate carrier system to significantly simplify the calculations. Since only processes within ultrashort pulses are considered, the TTM for SiO_2_ and PS is neglected.

Symbol	Unit	Au [28]	Si [25]	SiO_2_	PS
*G*	W/(m^−3^K)	2.1 × 10^16^	$C_{e}$/$\tau_{e}$	-	-
$C_{e}$	J/(m^−3^K)	$A_{e}$ $T_{e}$	3$n_{e}k_{B}$	-	-
$k_{e}$	W/(m K)	$KT_{e}$/$T_{l}$	$k_{e}$($T_{e}$)	-	-
$C_{l}$	J/(m^−3^K)	2.5 × 10^6^	$C_{l}$($T_{l}$)	-	-
$k_{l}$	W/(m K)	1 [29]	$k_{l}$($T_{l}$)	-	-

## Data Availability

The raw data supporting the conclusions of this article will be made available by the authors on request.
